# Supplementary material for: Structure and mechanism of the human NHE1-CHP1 complex
Source: Nat Commun. 2021 Jun 9;12:3474. doi: 10.1038/s41467-021-23496-z (PMC8190280; doi:10.1038/s41467-021-23496-z)
Supplement: Supplementary file 1 — Supplementary Information [file 41467_2021_23496_MOESM1_ESM.pdf]

Supplementary information for

Structure and mechanism of the human NHE1-CHP1 complex

Yanli Dong, Yiwei Gao, Alina Ilie, DuSik Kim, Annie Boucher, Bin Li, Xuejun C. Zhang, John Orlowski, and Yan Zhao

This file contains Supplementary Figure 1–9 and Supplementary Table 1–2.

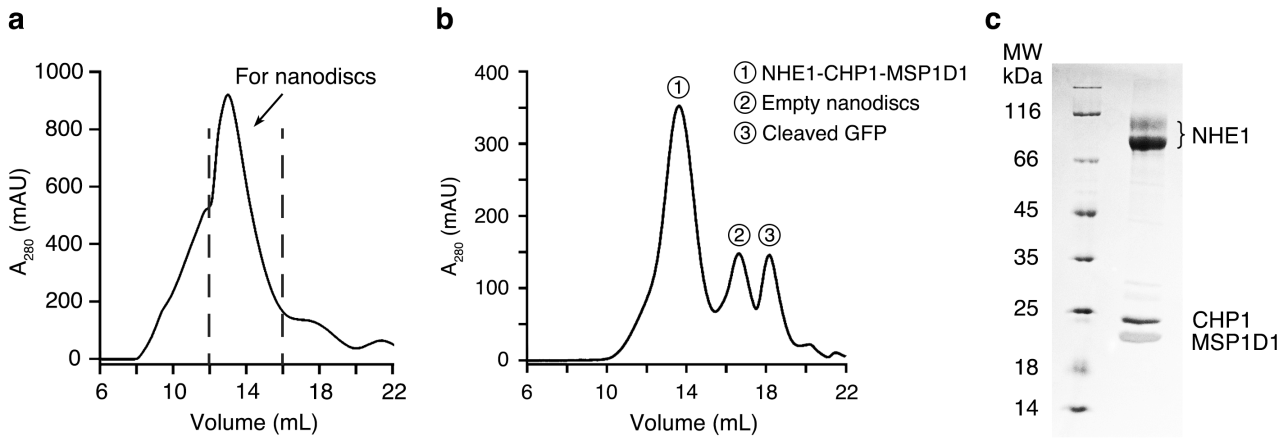

Supplementary Figure 1. Reconstitution of NHE1-CHP1 complex in nanodiscs.

a. Size-exclusion chromatogram (Superose 6 increase) of the purified sample of NHE1-CHP1 complex. Peak fractions (marked within black dashed lines) were used for nanodiscs reconstitution. **b.** Size-exclusion chromatogram of reconstituted NHE1-CHP1 complex into lipid nanodiscs. Peaks of NHE1-CHP1-MSP1D1 nanodiscs, empty nanodiscs, and cleaved GFP are indicated. **c.** Coomassie-blue-stained SDS-PAGE gel of the NHE1-CHP1-MSP1D1 nanodiscs. Components of the complex are labeled. NHE1 appeared in two bands due to different extent of glycosylation. The experiments were repeated independently with more than 3 times with similar results.

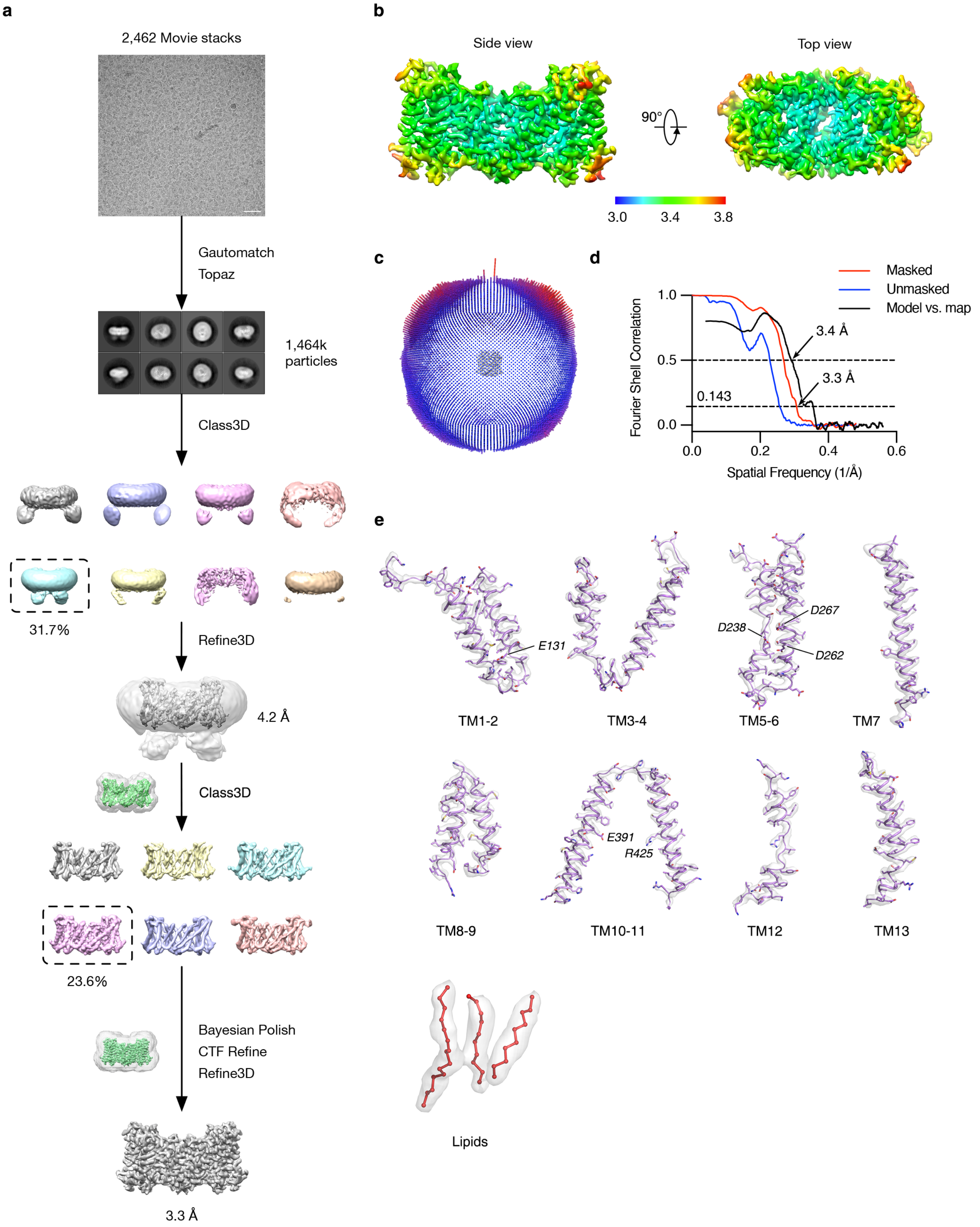

Supplementary Figure 2. Cryo-EM analysis of NHE1-CHP1$^{Na/7.5}$ complex.

a. Flow chart of cryo-EM data processing. A total of 2,462 movie stacks were collected (Bar = 40 nm). A representative motion-corrected micrograph of this dataset is shown here. Particles were picked using Gautomatch, Topaz, and Template Picker in cryoSPARC, and were then submitted to several rounds of 3D classification, Bayesian polish, and CTF and 3D-auto refinement. The resulting map was reported at 3.3 Å. The CHP1 density is weak due to conformational flexibility. The same map of the lower threshold (transparent) and the higher threshold were overlaid to show CHP1 and TM helices features, respectively. Masks used for 3D classification and refinement were also shown as transparent surfaces alongside the arrows. **b.** Sharpened map of the NHE1-CHP1$^{Na/7.5}$ complex, colored according to local resolution. **c.** Angular distribution of the particles contributing to the final reconstitution. The length of each spike indicates of the number of particles in the designated orientation. **d.** The half-map and model-map Fourier shell correlation (FSC) curves of NHE1-CHP1$^{Na/7.5}$ complex. **e.** Representative EM maps for the NHE1-CHP1$^{Na/7.5}$ complex as well as lipid molecules.

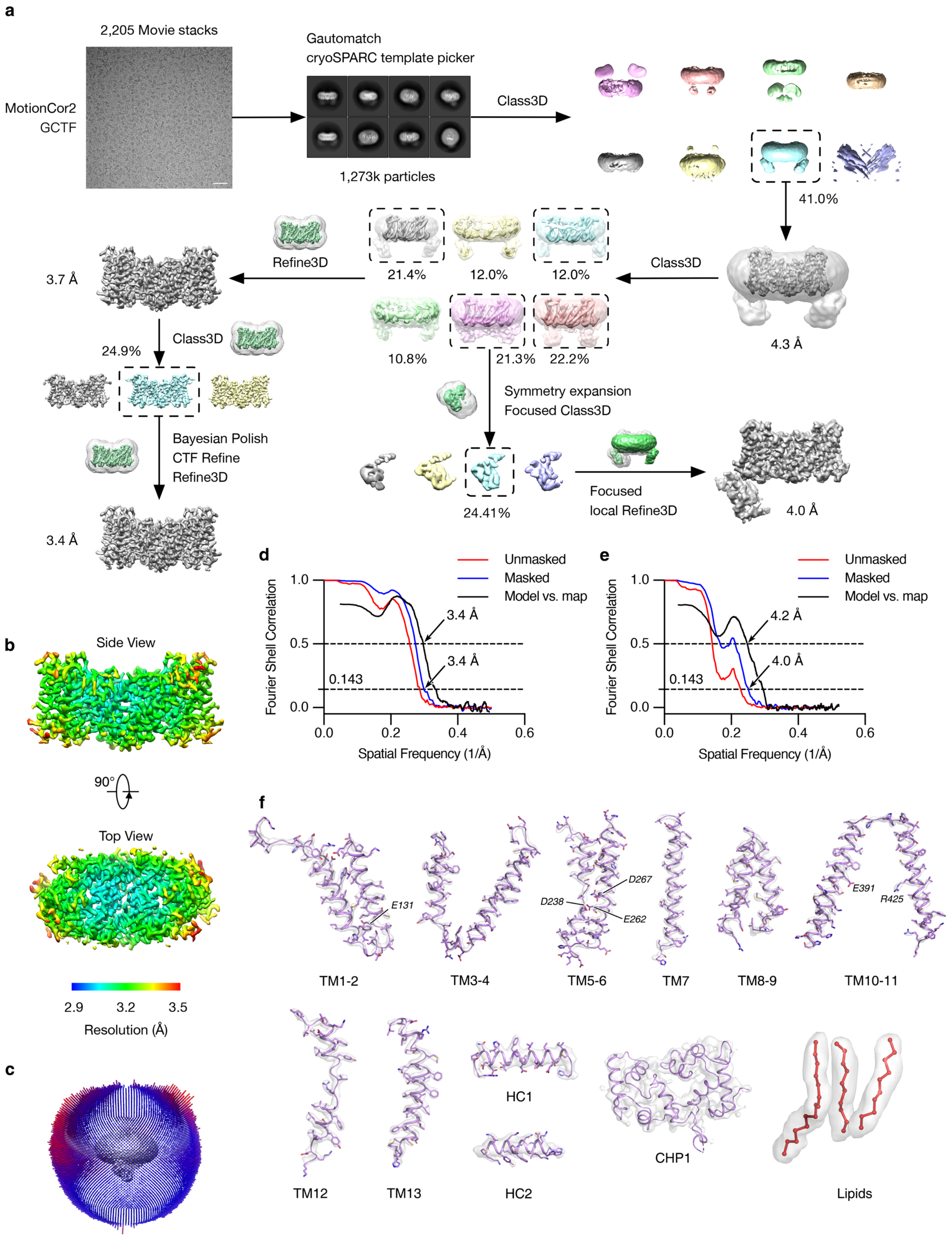

Supplementary Figure 3. Cryo-EM analysis of NHE1-CHP1$^{Na/6.5}$ complex.

a. Flow chart of cryo-EM data processing. A total of 2,205 movie stacks were collected and motion-corrected (Bar = 40 nm). A representative motion-corrected micrograph of this dataset is shown here. Particles were picked using Gautomatch and Template Picker in cryoSPARC. Junk particles were removed using several rounds of 2D and 3D classification. The NHE1-focused reconstruction yielded a map with a reported resolution of 3.4 Å. The CHP1-focused classification resulted in a 4.0-Å map, composed of the NHE1 dimer and one CHP1 molecule. The same maps of the lower threshold (transparent) and the higher threshold were overlaid to show CHP1 and TM helix features, respectively. Masks used for 3D classification and refinement were also shown as transparent surfaces alongside the arrows. **b.** Sharpened map of the NHE1-CHP1$^{Na/6.5}$ complex, colored according to local resolution estimation. **c**. Angular distribution of particles used in the final reconstruction. The length of each spike indicates of the number of particles in the designated orientation. **d–e.** FSC curves of the NHE1 dimer and NHE1-CHP1 complex, respectively, calculated between two independently refined half-maps before (red) and after (blue) post-processing, overlaid with an FSC curve calculated between the cryo-EM density map and the structural model shown in black. **f.** Representative EM maps for the NHE1-CHP1$^{Na/6.5}$ complex.

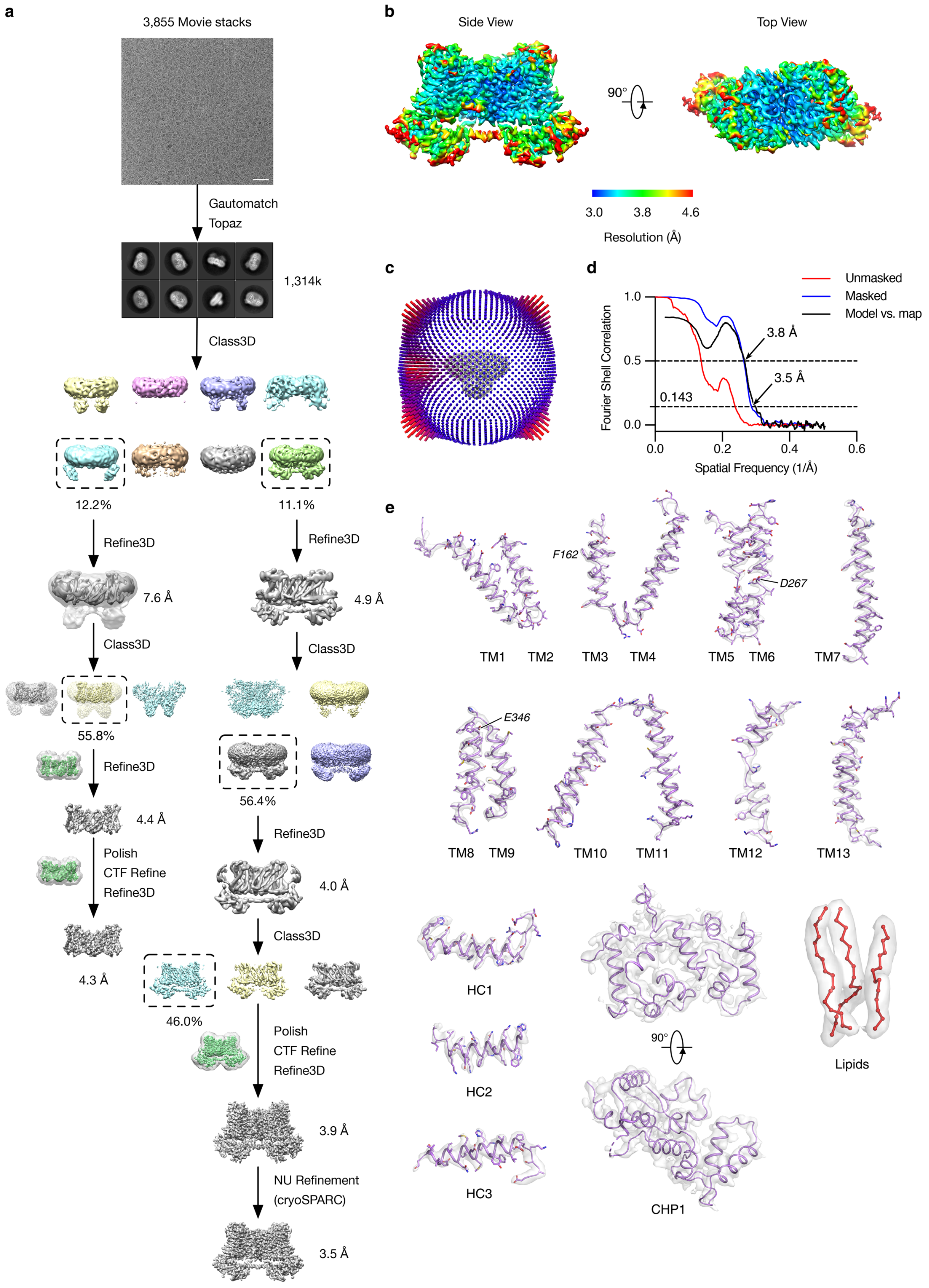

Supplementary Figure 4. Cryo-EM analysis of NHE1-CHP1$^{K/cariporide}$ complex.

a. Flow chart of cryo-EM data processing. A total of 3,855 movie stacks were collected (Bar = 40 nm). A representative motion-corrected micrograph of this dataset is shown here. Particles were picked using Gautomatch and Topaz, and was subjected to 3D classification. One of the resulting class displayed a discernable shape of CHP1 and was thus selected and submitted to further 3D classification, Bayesian Polish, CTF refinement and 3D auto refinement. The resulting map was reported at 3.6 Å. Masks used for 3D classification and refinement were shown as transparent surfaces alongside the arrows. **b.** Sharpened map of NHE1-CHP1$^{Na/7.5}$ complex, colored according to local resolutions. **c.** Angular distribution of the particles contributing to the final reconstitution. The length of each spike indicates of the number of particles in the designated orientation. **d.** The half-map and model-map FSC curves of the NHE1-CHP1$^{K/cariporide}$ complex. **e.** Representative EM maps for the NHE1-CHP1$^{Na/7.5}$ complex and lipid molecules.

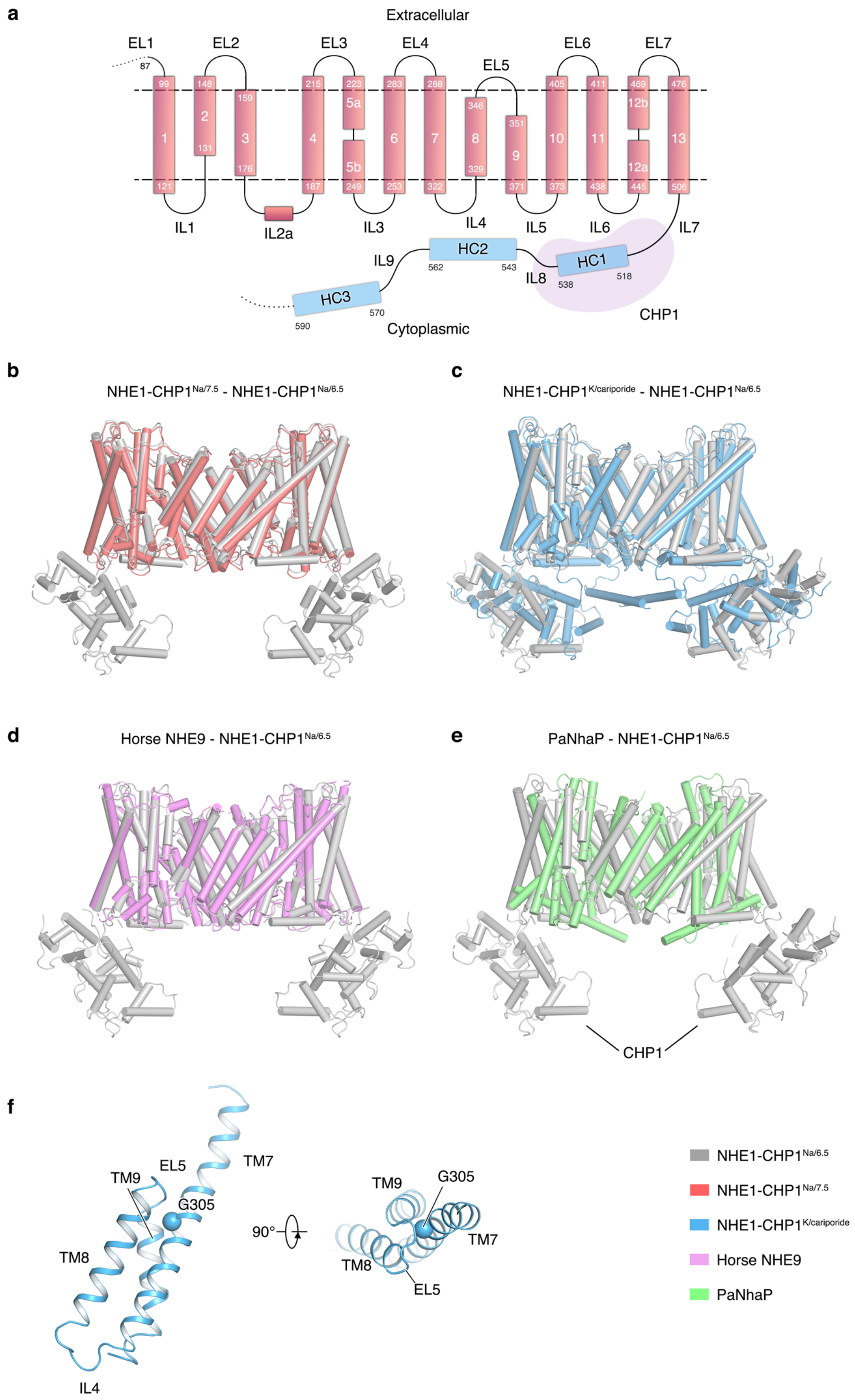

Supplementary Figure 5. Architecture of the NHE1-CHP1 complex.

a. A topology model of secondary structures of the NHE1-CHP1 complex. **b.** Superposition of the complex structures of NHE1-CHP1$^{Na/6.5}$ (grey) and NHE1-CHP1$^{Na/7.5}$ (red). **c.** Structural comparison of the NHE1-CHP1$^{K/cariporide}$ (blue) and NHE1-CHP1$^{Na/6.5}$ (grey) complexes. **d.** Structural comparison of the NHE1-CHP1$^{Na/6.5}$ and horse NHE9 (PDB ID: 6Z3Z), which colored in grey and magenta, respectively. **e.** Structural

comparison of the NHE1-CHP1$^{Na/6.5}$ complex with PaNhaP (PDB ID: 4CZA). The NHE1-CHP1$^{Na/6.5}$ complex and PaNhaP are colored in grey and green, respectively. **f.** TM helix bundle composing of TMs 7–9. G305 is shown as a sphere.

```
                    ----------------------------------------------------------------------------

hNHE1     1    ---------MVLRSGICGLSPHRIFPSLLVVVALVGLLPVLRSHGLQLSPTASTIRSSEPPRERSIGDVTTAPPEVTPESRPVNHSVTDH
hNHE2     1    --------MEPLGN---WRSLRAPLPPMLLLLLLQVAGPVGALAETLLNAPRAMGTSSSPPSPASVVAPGTTLFEESR------------
hNHE3     1    ---------------MWGLGARGPDRGLLLALALGGLARAG---------------GVEVEPGGAHGESGG-------------------
hNHE4     1    ---------MALQM---FVTY-SPWNCLLLLVALECSEASSDLNESAN--------STAQYASNAWFAAASSEPEEG-------------
hNHE5     1    ---------------MLRAALS------LLALPLAGAAEEP---------------TQKPESPGEP--PPG-------------------
hNHE6     1    MARRGWRRAPLRRGVGSSPRARRLMRPLWLLLAVGVFDWAGASD-GGGGEARAMDEEIVSEKQAEESHRQDSANLLIFILLLTLT-----
hNHE7     1    MEPGDAARPGSGRATGAPP-PRLLLLPLLLGWGLRVAAAASASSSGAAAEDSSAMEELATEKEAEESHRQDSVSLLTFILLLTLT-----
hNHE8     1    --------------------------------MGEKMAEEERFPNTTHEGFNVTLHTTLVVTTKLVLPTPGKPILPVQTG----------
hNHE9     1    MER---------------------------------------------------QSRVMSEKDEYQFQHQGAVELLVFNFLLILT-----
MjNhaP1   1    ------------------------------------------------------------------------------------------
PaNhaP    1    ------------------------------------------------------------------------------------------

               ---- EL1 TM1 IL1 TM2 EL2

hNHE1     82   GMKPRKAFPVLGIDYTHVRTPFEISLWILLACLMKIGFHVIPTISSIVPESCLLIVVGLLVGGLIKGVG-ETPP----------------
hNHE2     67   -------LPVFTLDYPHVQIPFEITLWILLASLAKIGFHLYHKLPTIVPESCLLIMVGLLLGGIIFGVDEKSPP----------------
hNHE3     41   -------FQVVTFEWAHVQDPYVIALWILVASLAKIGFHLSHKVTSVVPESALLIVLGLVLGGIVWAADHIASF----------------
hNHE4     56   -------ISVFELDYDYVQIPYEVTLWILLASLAKIGFHLYHRLPGLMPESCLLILVGALVGGIIFGTDHKSPP----------------
hNHE5     33   -------LELFRWQWHEVEAPYLVALWILVASLAKIVFHLSRKVTSLVPESCLLILLGLVLGGIVLAVAKKAEY----------------
hNHE6     84   ------ILTIWLFKHRRARFLHETGLAMIYGLLVGLVLRYGIHVPSDVNNVTLSCEVQS-SPTTLLV-----------------------
hNHE7     84   ------ILTIWLFKHRRVRFLHETGLAMIYGLIVGVILRYGTPATSGRD-KSLSCTQEDRAFSTLLVNVSGKFFEYTLKGEISPGKINSV
hNHE8     48   --------EQAQQEEQSSGMTIFFSLLVLAICIILVHLLIRYRLH-FLPESVAVVSLGILMGAVIKIIEFKKLAN---------------
hNHE9     34   ------ILTIWLFKNHRFRFLHETGGAMVYGLIMGLILRYATAPTDIESGTVYDCVKLTFSPSTLLVNITDQVYEYKYKREISQHNINPH
MjNhaP1   1    -----------------MELMMAIGYLGLALVLGSLVAKIAEKLK--IPDIPLLLLGLIIGPFLQIIPSDSAM-----------------
PaNhaP    1    -----------------MIELSLAEALFLILFTGVISMLISRRTG--ISYVPIFILTGLVIGPLLKLIPRDLAH----------------

               TM3 IL2a TM4 EL3 TM5a

hNHE1     154  ---------FLQSDVFFLFLLPPIILDAGYFLPLRQFTENLGTILIFAVVGTLWNAFFLGGLMYAVCLVG---GEQ-INNIGLLDNLLFG
hNHE2     134  ---------AMKTDVFFLYLLPPIVLDAGYFMPTRPFFENIGTIFWYAVVGTLWNSIGIGVSLFGICQIE---AFG-LSDITLLQNLLFG
hNHE3     108  ---------TLTPTVFFFYLLPPIVLDAGYFMPNRLFFGNLGTILLYAVVGTVWNAATTGLSLYGVFLSG---LMG-DLQIGLLDFLLFG
hNHE4     123  ---------VMDSSIYFLYLLPPIVLEGGYFMPTRPFFENIGSILWWAVLGALINALGIGLSLYLICQVK---AFG-LGDVNLLQNLLFG
hNHE5     100  ---------QLEPGTFFLFLLPPIVLDSGYFMPSRLFFDNLGAILTYAVVGTLWNAFTTGAALWGLQQAG---LVAPRVQAGLLDFLLFG
hNHE6     144  ---------TFDPEVFFNILLPPIIFYAGYSLKRRHFFRNLGSILAYAFLGTAISCFVIGSIMYGCVTLMKVTGQLA-GDFYFTDCLLFG
hNHE7     168  EQNDMLRKVTFDPEVFFNILLPPIIFHAGYSLKKRHFFRNLGSILAYAFLGTAVSCFIIGNLMYGVVKLMKIMGQLS-DKFYYTDCLFFG
hNHE8     114  ----WKEEEMFRPNMFFLLLLPPIIFESGYSLHKGNFFQNIGSITLFAVFGTAISAFVVGGGIYFLGQAD------VISKLNMTDSFAFG
hNHE9     119  QGNAILEKMTFDPEIFFNVLLPPIIFHAGYSLKKRHFFQNLGSILTYAFLGTAISCIVIGLIMYGFVKAMIHAGQLKNGDFHFTDCLFFG
MjNhaP1   55   ----------EIFEYAGPIGLIFILLGGAFTMRISLLKRVIKTVVRLDTITFLITLLISGFIFNMVLNLP----------YTSPVGYLFG
PaNhaP    55   ----------EIFDFVRVFGLVIILFTEGHNLSWRLLKKNMPTIVTLDTIGLILTALIAGFIFKVVFNS------------SFLLGFLFG

               TM5b IL3 TM6 EL4 TM7

hNHE1     232  SIISAVDPVAVLAVFEEIHINELLHILVFGESLLNDAVTVVLYHLFEEFANYE---------HVGIVDIFLGFLSFFVVALGGVLVGVVY
hNHE2     212  SLISAVDPVAVLAVFENIHVNEQLYILVFGESLLNDAVTVVLYNLFKSFCQMK---------TIETIDVFAGIANFFVVGIGGVLIGIFL
hNHE3     186  SLMAAVDPVAVLAVFEEVHVNEVLFIIVFGESLLNDAVTVVLYNVFESFVALGG-------DNVTGVDCVKGIVSFFVVSLGGTLVGVVF
hNHE4     201  SLISAVDPVAVLAVFEEARVNEQLYMMIFGEALLNDGITVVLYNMLIAFTKMHKF------EDIETVDILAGCARFIVVGLGGVLFGIVF
hNHE5     179  SLISAVDPVAVLAVFEEVHVNETLFIIVFGESLLNDAVTVVLYKVCNSFVEMGS-------ANVQATDYLKGVASLFVVSLGGAAVGLVF
hNHE6     225  AIVSATDPVTVLAIFHELQVDVELYALLFGESVLNDAVAIVLSSSIVAYQPAGDN-----SHTFDVTAMFKSIGIFLGIFSGSFAMGAAT
hNHE7     257  AIISATDPVTVLAIFNELHADVDLYALLFGESVLNDAVAIVLSSSIVAYQPAGLNTHA-----FDAAAFFKSVGIFLGIFSGSFTMGAVT
hNHE8     195  SLISAVDPVATIAIFNALHVDPVLNMLVFGESILNDAVSIVLTNTAEGLTRKNMSDVS------GWQTFLQALDYFLKMFFGSAALGTLT
hNHE9     209  SLMSATDPVTVLAIFHELHVDPDLYTLLFGESVLNDAVAIVLTYSISIYSPK-ENPNA-----FDAAAFFQSVGNFLGIFAGSFAMGSAY
MjNhaP1   126  AITAATDPATLIPVFSRVRTNPEVAITLEAESIFNDPLGIVSTSVILGLFG--------------LFSSSNPLIDLITLAGGAIVVGLLL
PaNhaP    124  AIIGATDPATLIPLFRQYRVKQDIETVIVTESIFNDPLGIVLTLIAISMLVPGYGGGIFSTLSEKLGIYAGGVIYFLYNVSVSISLGIFL

               TM7 IL4 TM8 EL5 TM9 IL5 TM10

hNHE1     313  GVIAAFTSRFTSH--IRVIEPLFVFLYSYMAYLSAELFH------LSGIMALIASGVVMRPYVEANI---------SHKSHTTIKYFLKM
hNHE2     293  GFIAAFTTRFTHN--IRVIEPLFVFLYSYLSYITAEMFH------LSGIMAITACAMTMNKYVEENV---------SQKSYTTIKYFMKM
hNHE3     269  AFLLSLVTRFTKH--VRIIEPGFVFIISYLSYLTSEMLS------LSAILAITFCGICCQKYVKANI---------SEQSATTVRYTMKM
hNHE4     285  GFISAFITRFTQN--ISAIEPLIVFMFSYLSYLAAETLY------LSGILAITACAVTMKKYVEENV---------SQTSYTTIKYFMKM
hNHE5     262  AFLLALTTRFTKR--VRIIEPLLVFLLAYAAYLTAEMAS------LSAILAVTMCGLGCKKYVEANI---------SHKSRTTVKYTMKT
hNHE6     310  GVVTALVTKFTKLREFQLLETGLFFLMSWSTFLLAEAWG------FTGVVAVLFCGITQAHYTYNNL---------STESQHRTKQLFEL
hNHE7     342  G-VNANVTKFTKLHCFPLLETALFFLMSWSTFLLAEACG------FTGVVAVLFCGITQAHYTYNNL---------SVESRSRTKQLFEV
hNHE8     279  GLISALVLKHIDLRKTPSLEFGMMIIFAYLPYGLAEGIS------LSGIMAILFSGIVMSHYTHHNL---------SPVTQILMQQTLRT
hNHE9     293  AIITALLTKFTKLCEFPMLETGLFFLLSWSAFLSAEAAG------LTGIVAVLFCGVTQAHYTYNNL---------SSDSKIRTKQLFEF
MjNhaP1   202  AKIYEKIIIHCDFHEY--VAPLVLGGAMLLLYVGDDLLPSICGYGFSGYMAVAIMGLYLGDALFRADD----------IDYKYIVSFCDD
PaNhaP    214  GILGYKFIKRTGIFDFPEIEAFSLSLAFLGFFIGERLD-------ASGYLVATVTGIVLGNYKLLKPRENIRILKRLQRAIEKEVHFNDT
```

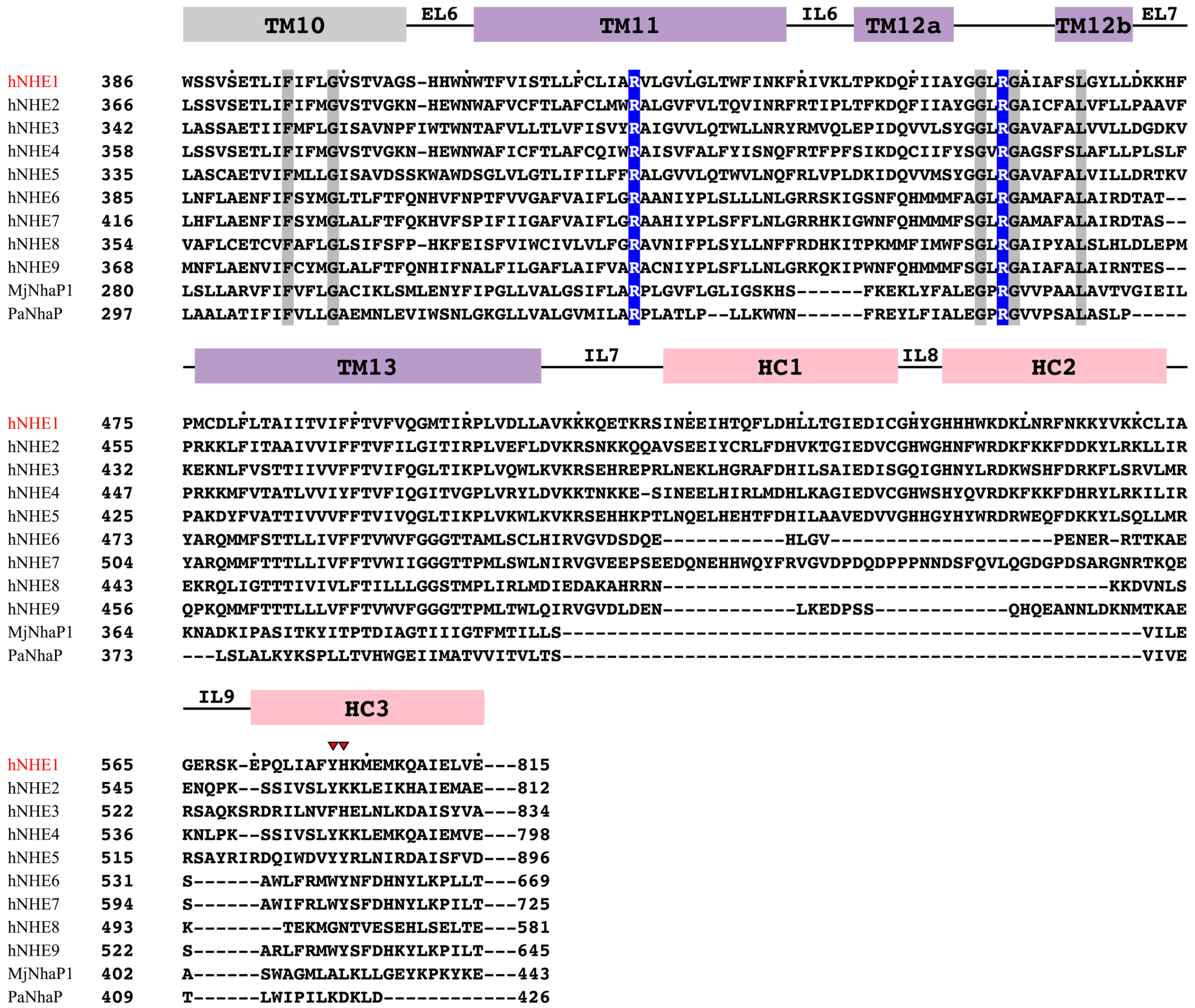

Supplementary Figure 6. Sequence alignment of sodium/proton exchangers.

Secondary structural elements of hNHE1 are marked above the sequence alignment, and unmodeled loops are represented as dashed lines. Helices of the dimerization domain, core domain, and from cytoplasmic region are colored in grey, purple, and pink. Mutation sites are marked as red triangles. Conservative residues among these proteins are highlighted in red, blue and grey for acidic, basic, and others, respectively.

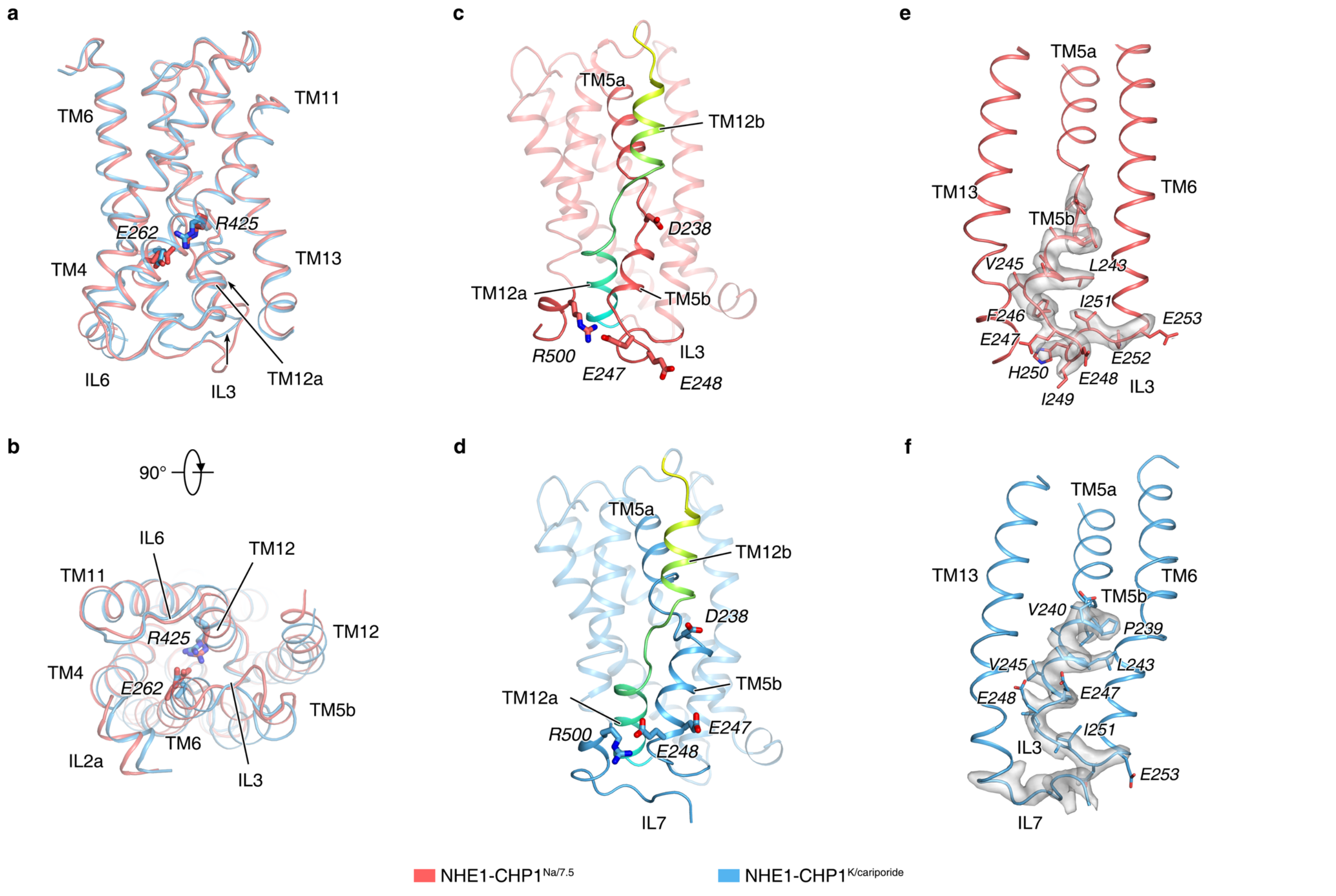

Supplementary Figure 7. Structural analysis of core domains.

a–b. Structural comparison of the core domains viewed parallel to the membrane and from the intracellular side of the membrane, respectively. The core domain of NHE1-CHP1$^{Na/7.5}$ complex and NHE1-CHP1$^{K/cariporide}$ complex are colored in red and blue, respectively. Sidechains of the E262 and R425 are shown in sticks. **c–d.** Closeup view of the model around TM5b and IL3 in the inward- and outward-facing conformations, respectively. Selected residues D238, D247, D248, and R500 are shown as sticks. TM12 is colored in a cyan-to-green spectrum. **e–f.** Local structures of TM5b, TM6, TM13, IL3, and IL7 in NHE-CHP1$^{Na/7.5}$ complex (**e**) and NHE1-CHP1$^{K/cariporide}$ (**f**). The corresponding densities of TM5b, IL3, and IL7 are shown in transparent grey surface.

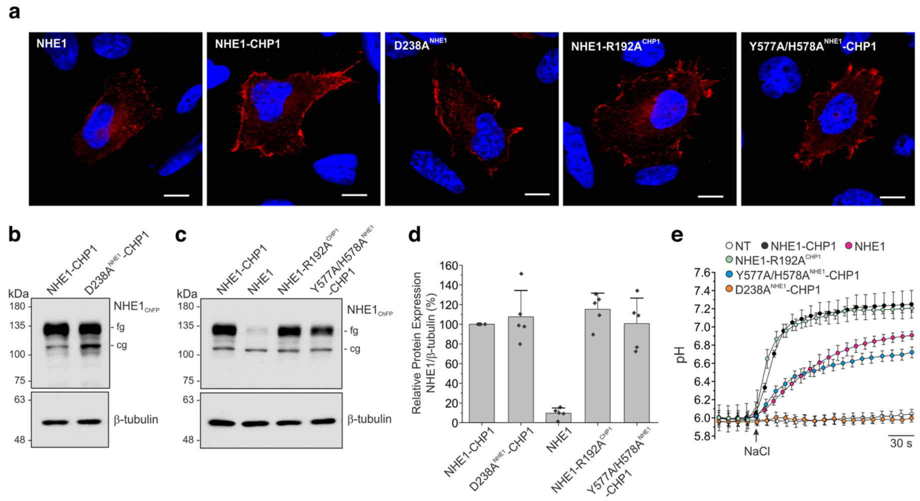

Supplementary Figure 8. Biochemical and functional analysis of NHE1-CHP1 complex.

a. Cellular distribution of NHE1 in AP-1 cells. AP-1 cells were transiently transfected (48 h) with wild-type NHE1 tagged at its C-terminus with mCherry fluorescent protein alone or wild type or mutant NHE1 co-expressed with either wild-type CHP1 or mutant CHP1 ($R192A^{CHP1}$), as indicated. Scale bars represent 10 µM. Images are representative of at least 6 different cells from two independent transfections. **b–c.** Assessment of protein expression of NHE1. Blots were stripped and reprobed with a mouse monoclonal anti-β-tubulin antibody to control for protein loading. Blots are representative images from five separate experiments. **d.** Quantitation of protein expression was determined by densitometry of X-ray films exposed within the linear range of the films. To control for subtle loading variations, NHE1 expression determined relative to endogenous β-tubulin (ratio of NHE1/β-tubulin) and then normalized to 100%. Values represent the mean ± S.D. (n = 5 independent blots). **e.** Measurement of cytoplasmic pH following an NH_4Cl-imposed acid-load in AP-1 cells transfected with NHE1 and CHP1. NHE1 activity was defined as the initial linear rate of pH recovery as a function of time upon reintroduction of Na^+-rich solution. Tracings are a representative experiment (mean ± S.E.M.; NT, n = 8 cells; NHE1, n = 3 cells, NHE1-CHP1, n = 4 cells; $D238A^{NHE1}$-CHP1, n = 4 cells; NHE1-$R192A^{CHP1}$, n = 4; $Y577A/H578A^{NHE1}$-CHP1, n = 5 cells) of 2 or 3 independent experiments. Source data are provided as a Source Data file.

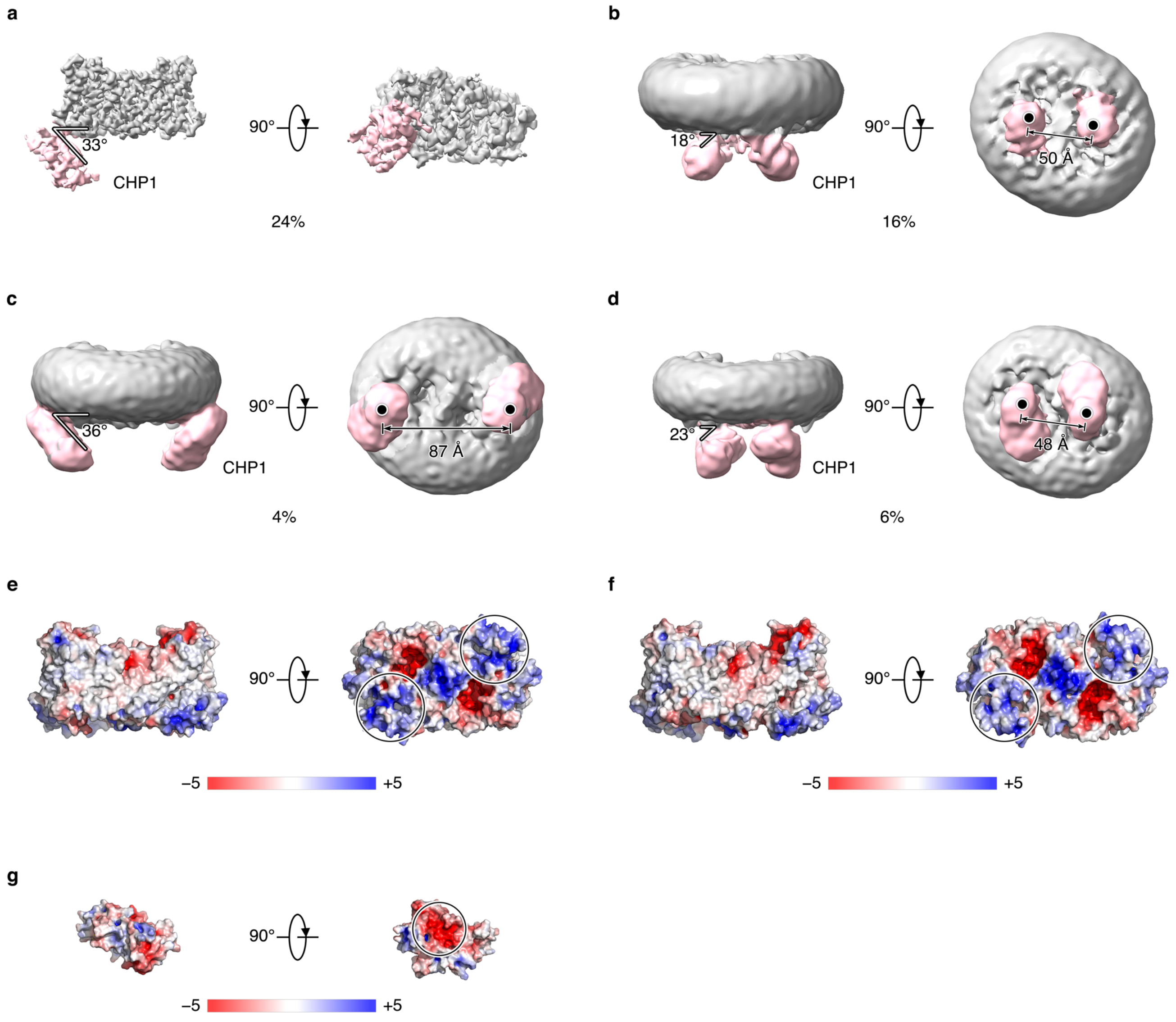

Supplementary Figure 9. Conformational heterogeneity and electrostatic potentials of the NHE1-CHP1 complex.

a–d. Representative conformations of CHP1 (pink) in the NHE1-CHP1$^{Na/6.5}$ complex (**a–b**) and NHE1-CHP1$^{Na/7.5}$ complex (**c–d**). **e–g**. The electrostatic potentials of NHE1 in NHE1-CHP1$^{Na/6.5}$ complex (**e**) and NHE1-CHP1$^{K/cariporide}$ complex (**f**). **g**. The electrostatic potential of CHP1. The NHE1 and CHP1 interaction areas are marked.

Supplementary Table 1. Cryo-EM data collection, processing and model validation.

	NHE1-CHP1$^{Na/7.5}$ complex	NHE1-CHP1$^{Na/6.5}$ complex	NHE1-CHP1$^{K/cariporide}$ complex
	Accession		
PDB ID	7DSW	7DSV	7DSX
EMDB Accession	EMD-30848	EMD-30847	EMD-30849
	Data Collection		
Microscope	Titan Krios	Talos Arctica	Titan Krios
Camera	K2 Summit	K2 Summit	K2 Summit
Voltage (kV)	300	200	300
Magnification	130,000 ×	130,000 ×	130,000 ×
Pixel Size (Å)	1.04	1	1.04
Defocus Range (μm)	−1.2 ~ −2.2	−1.2 ~ −2.2	−1.2 ~ −2.2
Energy Filter Slit Width (eV)	20	20	20
Total Dose (e/Å^2)	60	50	60
Micrographs (No.)	2,462	2,205	3,855
Final Particles (No.)	108,712	100,503	61,460
Map Resolution (Å)	3.3	3.4	3.5
	Model Validation		
Number of Atoms	6,736	6,768	10,982
Residues	840	840	1,370
Ligands	N/A	N/A	Cariporide
Sharpening B-factor (Å^2)	131	175	84
R. M. S. Deviations			
Bond Lengths (Å)	0.006	0.006	0.009
Bond Angles (°)	0.779	0.77	1.046
Ramachandran			
Favored (%)	91.87	92.34	91.16
Allowed (%)	8.13	7.66	8.84
Outlier (%)	0.00	0.00	0.00
MolProbity	2.27	2.11	2.31
EMRinger	1.35	1.54	1.96

Supplementary Table 2. Primers used in this study.

Name	Sequence
NHE1-H-SalI-F	ATACGCGTCGACATGGTTCTGCGGTCTGGCAT
NHE1-H-NotI-R	AAATATGCGGCCGCCTGCCCCTTGGGGAAGGACG
CHP1-H-pET21a-F	CTGGTGGACAGCAAATGGGTCGCGGATCCGAATTCATGGGTTCTCGGGCCTCCA
CHP1-H-pET21a-R	TCTCAGTGGTGGTGGTGGTGGTGATGATGCTCGAGGTGAAGAAATCGGATGCTCA
CHP1-H-SalI-F	ATACGCGTCGACATGGGTTCTCGGGCCTCCA
CHP1-H-NotI-R	AAATATGCGGCCGCCTGCCCCTTGGGGAAGGACG
NHE1-CHP1-biGBac1a-D238A^{NHE1}-F	AGCATCATCTCGGCCGTGGCACCCGTGGCGGTTCTGGCTGTCTTT
NHE1-CHP1-biGBac1a-D238A^{NHE1}-R	AAAGACAGCCAGAACCGCCACGGGTGCCACGGCCGAGATGATGCT
NHE1-CHP1-biGBac1a-Y577A/H578A^{NHE1}-F	GAGCCCCAGCTCATTGCCTTCGCCGCCAAGATGGAGATGAAGCAGGCCATCG
NHE1-CHP1-biGBac1a-Y577A/H578A^{NHE1}-R	CGATGGCCTGCTTCATCTCCATCTTGGCGGCGAAGGCAATGAGCTGGGGCTC
NHE1-CHP1-biGBac1a-R192A^{CHP1}-F	GTAGAACAGAAAATGAGCATCGCATTTCTTCACTGATCTAG
NHE1-CHP1-biGBac1a-R192A^{CHP1}-R	CTAGATCAGTGAAGAAATGCGATGCTCATTTTCTGTTCTAC
