# Supplementary material for: Structure and mechanism of the human NHE1-CHP1 complex
Source: Nat Commun. 2021 Jun 9;12:3474. doi: 10.1038/s41467-021-23496-z (PMC8190280; doi:10.1038/s41467-021-23496-z)
Supplement: Supplementary file 3 — Reporting Summary [file 41467_2021_23496_MOESM3_ESM.pdf]

nature research

Corresponding author(s): Yan Zhao, John Orlowski, Xuejun C. Zhang

Last updated by author(s): Apr 18, 2021

Reporting Summary

Nature Research wishes to improve the reproducibility of the work that we publish. This form provides structure for consistency and transparency in reporting. For further information on Nature Research policies, see our Editorial Policies and the Editorial Policy Checklist.

Statistics

For all statistical analyses, confirm that the following items are present in the figure legend, table legend, main text, or Methods section.

n/a	Confirmed	
☐	☒	The exact sample size (*n*) for each experimental group/condition, given as a discrete number and unit of measurement
☐	☒	A statement on whether measurements were taken from distinct samples or whether the same sample was measured repeatedly
☐	☒	The statistical test(s) used AND whether they are one- or two-sided<br>*Only common tests should be described solely by name; describe more complex techniques in the Methods section.*
☒	☐	A description of all covariates tested
☒	☐	A description of any assumptions or corrections, such as tests of normality and adjustment for multiple comparisons
☐	☒	A full description of the statistical parameters including central tendency (e.g. means) or other basic estimates (e.g. regression coefficient) AND variation (e.g. standard deviation) or associated estimates of uncertainty (e.g. confidence intervals)
☐	☒	For null hypothesis testing, the test statistic (e.g. *F*, *t*, *r*) with confidence intervals, effect sizes, degrees of freedom and *P* value noted<br>*Give P values as exact values whenever suitable.*
☒	☐	For Bayesian analysis, information on the choice of priors and Markov chain Monte Carlo settings
☒	☐	For hierarchical and complex designs, identification of the appropriate level for tests and full reporting of outcomes
☒	☐	Estimates of effect sizes (e.g. Cohen's *d*, Pearson's *r*), indicating how they were calculated

Our web collection on statistics for biologists contains articles on many of the points above.

Software and code

Policy information about availability of computer code

Data collection	SerialEM 3.6
Data analysis	MotionCor2 1.3.2, GCTF 1.06, Gautomatch 0.56, cryoSPARC 2.15, RELION-3.1, UCSF Chimera 1.14, UCSF ChimeraX 1.1, PyMOL 2.4.0, PHENIX 1.16, Coot 0.8.9, OriginPro 2020 SR1 9.7.0.188

For manuscripts utilizing custom algorithms or software that are central to the research but not yet described in published literature, software must be made available to editors and reviewers. We strongly encourage code deposition in a community repository (e.g. GitHub). See the Nature Research guidelines for submitting code & software for further information.

Data

Policy information about availability of data

All manuscripts must include a data availability statement. This statement should provide the following information, where applicable:

- Accession codes, unique identifiers, or web links for publicly available datasets
- A list of figures that have associated raw data
- A description of any restrictions on data availability

The UniProtKB accession codes for NHE1 and CHP1 are listed as follows:
NHE1: P19634 [https://www.uniprot.org/uniprot/P19634]
CHP1: Q99653 [https://www.uniprot.org/uniprot/Q99653]

Structural data have been deposited in the Protein Data Bank (PDB) and the Electron Microscopy Data Bank (EMDB). The PDB ID and EMDB code for novel structures in this article are listed as follows:

NHE1-CHP1 Na/7.5 complex: 7DSW [http://doi.org/10.2210/pdb7DSW/pdb], EMD-30848 [https://www.emdataresource.org/EMD-30848]
NHE1-CHP1 Na/6.5 complex: 7DSV [http://doi.org/10.2210/pdb7DSV/pdb], EMD-30847 [https://www.emdataresource.org/EMD-30847]
NHE1-CHP1 K/cariporide complex: 7DSX [http://doi.org/10.2210/pdb7DSX/pdb], EMD-30849 [https://www.emdataresource.org/EMD-30849]

The source data for Figure 5f and Extended Data Figure 8 is provided as a Source Data File.

Field-specific reporting

Please select the one below that is the best fit for your research. If you are not sure, read the appropriate sections before making your selection.

☒ Life sciences ☐ Behavioural & social sciences ☐ Ecological, evolutionary & environmental sciences

For a reference copy of the document with all sections, see nature.com/documents/nr-reporting-summary-flat.pdf

Life sciences study design

All studies must disclose on these points even when the disclosure is negative.

Sample size

For functional assays of NHE1 activity, the appropriate sample size was estimated using the Resource Equation method (Mead R. 1988. "The design of experiments : statistical principles for practical applications". Cambridge, New York: Cambridge University Press). All the parameters in the equation use the degrees of freedom of the number of their concepts which is suitable for experiments that can be analyzed using the analysis of variance (ANOVA). This method is applicable when parameters such as expected standard deviations or expected differences in values between groups are unknown or very hard to estimate.

Data exclusions

For measurements of pH recovery, all data were included in the raw data Excel file. For statistical analyses, all data for each treatment group were analyzed by one-way ANOVA with the exception of the non-transfected (NT) AP-1 cell treatment group, where only 10 of 13 measurements were included. The selection of values for the NT group was random and performed to limit sample size variation between the different treatment groups for the ANOVA analysis which varied between 6 and 10 samples. Including all NT values in the ANOVA however did not affect the statistical outcome.

Replication

All attempts at replication were successful.

Randomization

All samples were segregated into groups according to treatment (i.e., expression of NHE1 wild-type and mutant constructs). Co-variants such as the total level of expression for NHE1 in each treatment group was assessed by western blots analysis.

Blinding

Blinding was not relevant in this study as the parameters being measured did not require subjective assessments of the treatments or their outcomes that might otherwise influence the validity of the results.

Reporting for specific materials, systems and methods

We require information from authors about some types of materials, experimental systems and methods used in many studies. Here, indicate whether each material, system or method listed is relevant to your study. If you are not sure if a list item applies to your research, read the appropriate section before selecting a response.

Materials & experimental systems

n/a	Involved in the study	
☐	☒	Antibodies
☐	☒	Eukaryotic cell lines
☒	☐	Palaeontology and archaeology
☒	☐	Animals and other organisms
☒	☐	Human research participants
☒	☐	Clinical data
☒	☐	Dual use research of concern

Methods

n/a	Involved in the study	
☒	☐	ChIP-seq
☒	☐	Flow cytometry
☒	☐	MRI-based neuroimaging

Antibodies

Antibodies used

(1) Anti-Na+/H+ exchanger-1 carboxy-terminus mouse monoclonal antibody, clone 4E9, (EMD Millipore Corp., catalog # MAB3140, Lot number 3521104).
(2) Anti-β-tubulin mouse monoclonal antibody, clone D66 (Millipore SigmaAldrich, purified from hybridoma cell culture, catalog # T0198, Lot number 035M4782V).
(3) Peroxidase-conjugated Affinity Pure F(ab)2 Fragment Goat Anti-Mouse IgG (H+L) (Jackson ImmunoResearch, catalog number 115-036-062; Lot number 136707).

Validation

(1) Anti-Na+/H+ exchanger-1 mouse monoclonal antibody, clone 4E9, has been published (Chahine, M., et al. (2005). J. Mol. Cell. Cardiol. 38(4):571-582) and validated for use in western blotting as indicated on manufacturer's website. Exhibits cross-reactivity for all vertebrates including fish, amphibians, birds, and mammals including humans.

(2) Anti-β-tubulin mouse monoclonal antibody, clone D66, has been published and validated for use in western blotting as indicated on manufacturer's website. Exhibits cross-reactivity for monkey, rat, mouse, chicken, sea urchin, rabbit, canine, human, bovine, and hamster.
(3) Peroxidase-conjugated Affinity Pure F(ab)2 Fragment Goat Anti-Mouse IgG (H+L) has been published and validated for use in immunohisto/cytochemistry, ELISA and western blotting as indicated on manufacturer's website. Antibody reacts with whole molecule mouse IgG based on immunoelectrophoresis and/or ELISA. It also reacts with the light chains of other mouse immunoglobulins. No antibody was detected against non-immunoglobulin serum proteins. The antibody has been tested by ELISA and/or solid-phase adsorbed to ensure minimal cross-reaction with human, bovine, and horse serum proteins, but it may cross-react with immunoglobulins from other species.

Eukaryotic cell lines

Policy information about cell lines

Cell line source(s)	HEK293F (R79-007, Gibco, USA), Chinese hamster ovary (CHO)-derived AP-1 cells (Rotin, D. and S. Grinstein (1989). "Impaired cell volume regulation in Na+-H+ exchange-deficient mutants." Am. J. Physiol 257(6 Pt 1): C1158-C1165.)
Authentication	Not authenticated.
Mycoplasma contamination	HEK293F cells were not tested for mycoplasma contamination. CHO/AP-1 cells were routinely tested for mycoplasma contamination and treated if detected.
Commonly misidentified lines (See ICLAC register)	No commonly misidentified cell lines were used in this study.
