# Supplementary figures and images for: Structure and mechanism of the human NHE1-CHP1 complex
Source: Nat Commun. 2021 Jun 9;12:3474. doi: 10.1038/s41467-021-23496-z (PMC8190280; doi:10.1038/s41467-021-23496-z)

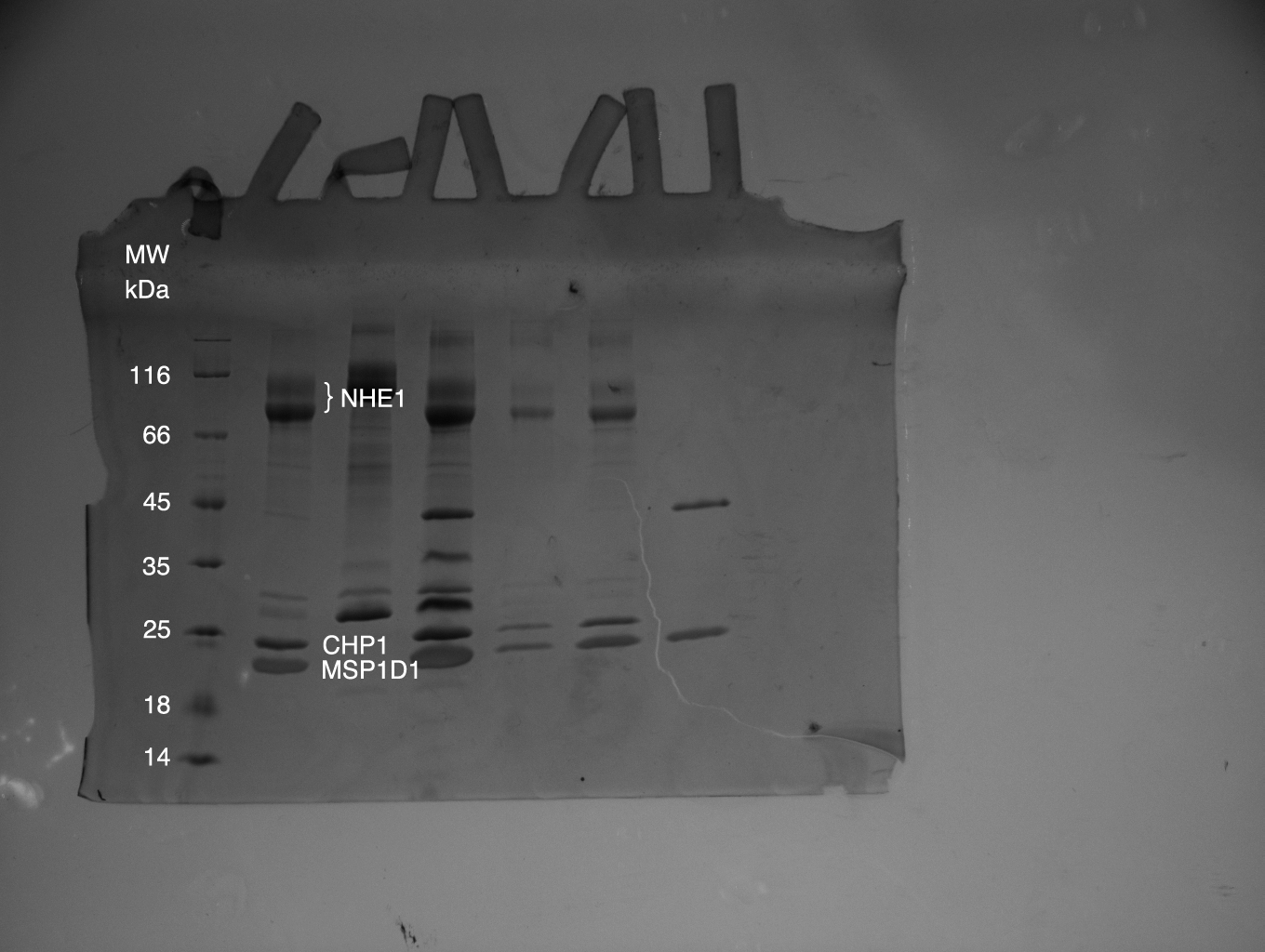

Supplement: Supplementary file 4 — Source Data [file 41467_2021_23496_MOESM4_ESM.zip › Source Data Files/Supplementary_Fig_1c-Protein_Purification.tif]
